# Deletion of N-acetylmuramyl-L-alanine amidases alters the host immune response to *Mycobacterium tuberculosis* infection

**DOI:** 10.1080/21505594.2021.1914448

**Published:** 2021-05-13

**Authors:** Nathan Scott Kieswetter, Mumin Ozturk, Shelby-Sara Jones, Sibusiso Senzani, Melissa Dalcina Chengalroyen, Frank Brombacher, Bavesh Kana, Reto Guler

**Affiliations:** a Cape Town Component, International Centre for Genetic Engineering and Biotechnology, Cape Town, South Africa; b Department of Pathology, Institute of Infectious Diseases and Molecular Medicine (IDM), Division of Immunology and South African Medical Research Council (SAMRC) Immunology of Infectious Diseases, Faculty of Health Sciences, University of Cape Town, Cape Town, South Africa; c DST/NRF Centre of Excellence for Biomedical TB Research, Faculty of Health Sciences, National Health Laboratory Service, University of the Witwatersrand, Johannesburg, South Africa; d Wellcome Centre for Infectious Diseases Research in Africa (CIDRI-Africa), Institute of Infectious Disease and Molecular Medicine (IDM), Faculty of Health Sciences, University of Cape Town, Cape Town, South Africa

**Keywords:** *Mycobacterium tuberculosis*, amidase, Ami1, Ami4, immunopathology, mice, macrophage, host–pathogen interaction, mutant

## Abstract

Peptidoglycan (PG), a heteropolysaccharide component of the mycobacterial cell wall can be shed during tuberculosis infection with immunomodulatory consequences. As such, changes in PG structure are expected to have important implications on disease progression and host responses during infection with *Mycobacterium tuberculosis*. Mycobacterial amidases have important roles in remodeling of PG during cell division and are implicated in susceptibility to antibiotics. However, their role in modulating host immunity remains unknown. We assessed the bacterial burden and host immune responses to *M. tuberculosis* mutants defective for either one of two PG N-acetylmuramyl-L-alanine amidases, Ami1 and Ami4, in bone marrow-derived macrophages (BMDM) and C57BL/6 mice. In infected BMDM, the single deletion of both genes resulted in increased proinflammatory cytokine responses. In mice, infection with the Δ*ami1* mutant led to differential induction of pro-inflammatory cytokines and chemokines, decreased cellular recruitment and reduced lung pathology during the acute phase of the infection. While increased proinflammatory cytokines production was observed in BMDM infected with the Δ*ami4* mutant, these effects did not prevail in mice. Infection using the Δ*ami1* and Δ*ami4* Mtb mutants showed that these genes are dispensable for intracellular mycobacterial growth in macrophages and mycobacterial burden in mice. These findings suggest that both Ami1 and Ami4 in *M. tuberculosis* are not essential for mycobacterial growth within the host. In summary, we show that amidases are important for modulating host immunity during Mtb infection in murine macrophages and mice.

## Introduction

Tuberculosis (TB) causes the largest number of human deaths attributable to a single bacterial human pathogen [1]. It is estimated that globally 1 in 4 people are infected with *Mycobacterium tuberculosis* (Mtb), the causative agent of TB [1,2]. The rapid emergence of drug-resistant TB and the continued failure to create a universally efficacious vaccine have highlighted the need for new effective therapies. The discovery and characterization of novel targets for therapeutic intervention thus remain a key area of TB research. The biosynthesis of the bacterial cell wall and remodeling of the complex macromolecules which comprise it are essential processes under strict regulatory control to facilitate normal cell division, growth, virulence, as well as resistance to antibiotics [3–5]. This dynamic balance of cell wall biogenesis vs. degradation is achieved via the coordinated action of multiple enzymes and dysregulation thereof has deleterious implications for the mycobacterial cell, an effect that can potentially inform novel drug design [6–9]. In the past, such a strategy has served as the basis for the successful development of many commercial antibiotics and similar approaches may be beneficial for TB [10].

Peptidoglycan (PG) is unique to the bacterial cell wall and essential for the maintenance of cytoplasmic turgor and cell morphology. PG is made up of crosslinked N‑acetylglucosamine (NAG) and N‑acetylmuramic acid (MurNAc or NAM), which are potent bacterial antigens that are detected by multiple host pattern recognition receptors (PRR) during infection [11]. Four PG recognition proteins (PGLYRP1-4), which directly bind to the PG of the cell wall have been identified in mammals. This detection is facilitated via recognition of the muramyl pentapeptide or tetrapeptide present on the bacterial cell wall [12]. Cytosolic nucleotide-binding oligomerization domain-containing protein 1 (NOD1) and nucleotide-binding oligomerization domain-containing protein 2 (NOD2) are the two most well-understood sensors of PG [11]. NOD1 identifies PG through the binding of the muropeptide γ-d-glutamyl-meso-diaminopimelic acid (iE‑DAP) [13,14]. NOD2 identifies PG via muramyl dipeptide (MDP), which is formed via the acetylation of MurNAc [15,16]. The binding of these ligands to either NOD1 or NOD2 ultimately results in the activation of two pathways, namely; the inhibitor of nuclear factor-κB (IKK) complex leading to activation of nuclear factor-κB (NF‑κB), or activation of transforming growth factor beta-activated kinase 1 (TAK1) and the mitogen-activated protein kinase (MAPK) signaling cascade [17]. Both of these pathways initiate the production of proinflammatory cytokines and chemokines which are essential for the host immune response and antigen presentation [17]. Additionally, NOD-, leucine-rich repeat receptor (LRR-) and pyrin domain-containing 3 (NLRP3) have also been implicated in the sensing of PG [18], whereas the role of toll-like receptors (TLR) in PG recognition remains controversial.

Bacteria have evolved several strategies to subvert immune detection to elicit chronic infection and prolonged survival. Often, this is facilitated via the remodeling of the PG sacculus. Previous studies in model pathogenic bacterial species have demonstrated that these organisms can evade the host immune system and enhance their pathogenicity through the deacetylation or the acetylation of the PG sugar backbone via the enzymes, N-acetylglucosamine-deacetylase A and O‑acetyltransferase, respectively [19–21]. More specifically, the deacetylation of NAG or MurNAc has been shown to abrogate NOD1/2 and inflammasome activation [18]. Additionally, Mtb can evade the NOD1 host immune response via the amidation of the meso–diaminopimelic acid (mDAP), thus removing the substrate required for the formation of iE-DAP, the ligand of NOD1 [22,23].

During growth and division, PG is remodeled via the action of several remodeling enzymes such as transpeptidases, endopeptidases, carboxypeptidases, amidases, and transglycosylases [6,24,25]. These enzymes have important roles in the hydrolysis, synthesis, crosslinking and remodeling of the PG sacculus and also mediate repair of damaged cell wall material [24,26]. In this study, we investigated the role of two N-acetylmuramoyl-L-alanine amidases (Ami1 and Ami4) in Mtb. These enzymes are important for PG degradation during cell division, PG recycling and homeostatic regulation of osmotic pressure via the hydrolysis of the bond between the glycan strand and stem peptide [26]. The crystal structure of Rv3717 (herein referred to as Ami1), a known mycobacterial amidase, has been explored and shown to bear structural similarity to the amidase-3 domain and containing amidases observed in *E. coli*, with demonstrated ability to cleave PG [27,28]. Whilst lacking efficient hydrolase activity relative to Ami1, Ami2 (CwlM, Rv3915) is essential in regulating cell growth via phospho-relay mechanisms [29,30]. In addition to Ami1 and Ami2, two more amidase-2 domain-containing amidases have been identified in Mtb, designated Ami3 (Rv3811) and Ami4 (Rv3594) [31]. In *Mycobacterium smegmatis*, Ami3 is stabilized by mannosylation and subject to degradation by the high-temperature requirement A (HtrA) protease as dysregulated levels of this protein resulted in cell death [9]. Ami1 deletion in *M. smegmatis* was found to confer dysfunctional septation, resulting in the formation of cellular chains, with a two-to-four-fold increase in susceptibility to a range of cell wall targeting antimicrobial agents [32]. In Mtb, an Ami1-deficient mutant is defective for survival in chronic infection in mice where Ami1 appears to play a synergistic role with RipA, another cell wall endopeptidase in mediating survival and tolerance [33]. In this study, we further investigate the effects of Mtb Ami1 and Ami4 defective mutants in primary murine macrophages as well as various *in vivo* host immune responses using C57BL/6 mice.

## Materials and methods

### Mice

Male wild-type C57BL/6 mice (Jackson Labs), aged between 8–12 weeks, were utilized for amidase infection studies as well as for the generation of BMDM. Mice were housed in a biosafety level 3 containment facility, maximum six per individually ventilated cage with filter tops (type 2 long), as well as dried wood shavings and shredded filter paper as floor coverings.

### Ethical statement

All experiments were conducted in accordance with the Animal Research Ethics Committee of South African National Standard (SANS 10386:2008) and the University of Cape Town, South Africa for practice on animal procedures. The protocol (Permit number: AEC 015/40 and AEC 015/36) was approved by the Animal Ethics Committee, Faculty of Health Sciences, University of Cape Town, South Africa.

### *Generation of BMDM and* in vitro *infection*

The generation of murine BMDM was achieved through bone marrow harvest and subsequent differentiation and polarization of murine pluripotent stem cells as previously described [34]. Post differentiation, BMDM were cultured in standard Dulbecco’s Modified Eagle Medium (DMEM) supplemented with 10% fetal calf serum (FCS) at 37°C. Cells were adhered over 12–24 hours in 96-well plates (Nunc, Denmark) at a concentration of 2 × 10^5^ cells per well. Post adherence, the cell media was supplemented with 100 U/mL recombinant IFN-γ (BD Biosciences) for 24 hours. Cells were infected with Mtb strains at a multiplicity of infection (MOI) 1 for indicated time points.

### Mycobacterium tuberculosis *strains and CFU analysis*

The Mtb deletion mutants (*Δami1, Δami4*) and complemented strains (*Δami1::ami1, Δami4::ami4*) were constructed from wild-type H37Rv (ATCC 25618) as described below using the primers detailed in Table S1 and S2. Cells were cultured at 37°C in 7H9 medium supplemented with 10% Middlebrook OADC Growth Supplement OADC and glycerol (1%) to an OD of ~0.6. To ensure single-cell stocks, the Mtb culture was vortexed vigorously using sterile glass beads. The culture was then centrifuged at 700 rpm for 5 min. The upper clump-free volume was removed to a new tube and stored at −80°C in 15% glycerol. To determine CFU, infected cells were lysed in 10% Triton X-100 (Sigma) and the cell lysate was plated on 7H11 agar supplemented with 10% Middlebrook OADC Growth Supplement and 0.5% glycerol. Plates were cultured for 2–3 weeks at 37°C.

### *Construction of amidase deficient and ami-complemented strains in* M. tuberculosis

To generate the *ami1* and *ami4* deletion mutants, H37*ami1*KO and H37*ami4*KO primers listed in Table S1 were designed to amplify the upstream and downstream regions of homology for both the *ami1* and *ami4* gene. The resulting upstream and downstream products were fused to yield *ami1* and *ami4* in-frame deletion alleles. These were then cloned into p2NIL [35] respectively, followed by insertion of a marker cassette from pGOAL17 [35] into the resulting vectors to create the final amidase deletion constructs, p2H37ΔAmi1G17 and p2H37ΔAmi4G17. These suicide vectors were then transformed into electro-competent *M. tuberculosis* H37Rv, which were then subjected to two-step allelic exchange mutagenesis yielding the Δ*ami1* and Δ*ami4* mutant strains. The resulting mutants were screened by PCR using the H37*ami1*pMV and H37*ami4*pMV then further validated using southern hybridization. To construct the complemented amidase strains used in this study, PCR amplification was conducted to amplify *ami1* and *ami4* genes and 400 bp upstream the transcriptional start codon. The fragments were then cloned into pMV306 (H) and clones were screened by restriction with BamHI. A single positive clone was then selected, and the genetic integrity of the vector was confirmed by extensive restriction profiling. The genetic integrity of the vector was then further confirmed by sequencing of the cloned region, which revealed that no mutations had occurred during PCR and cloning processes (data not shown). These vectors were then electroporated into the Δ*ami1* and Δ*ami4* mutant strains detailed above to create the Δ*ami1::ami1* and Δ*ami4::ami1* complemented strains, respectively.

### Genotyping and confirmation of amidase deletion

Mtb amidase deficient mutant strains were assessed via polymerase chain reaction (PCR). Mtb mutant stocks were streaked on 7H11 agar plates and incubated at 37°C for 4 weeks. A single colony was then isolated and inoculated in 50 µL sdH_2_O and heat-inactivated at 95°C for 10 min. A total of 25 µL chloroform was added, and the solution was centrifuged at maximum speed for 10 minutes. The top layer was used for standard PCR. The primers used for verification of the genotypes were as follows: Ami1 SC F 5ʹ- TGGACCTACGAGTTGGCC-3ʹ, Ami1 SC R1 5ʹ- GCCGAGTAGTTGACGTGGA-3ʹ, Ami1 SC R2 5ʹ- CCCTAGTCCTCGACAACTGC-3ʹ, Ami4 SC F 5ʹ- AGGCATCCGGAGGTATCC-3ʹ, Ami4 SC R1 5ʹ- CACTCGACGCCAATCATGT-3ʹ and Ami4 SC R2 5ʹ- CCGGTTGACATCGTTGCA-3ʹ. The cycling conditions are as follows: 95°C for 4 min followed by 35 cycles of 95°C for 30 secs, 60°C for 30 secs and 72°C for 1 min and a final 72°C for 7 min step. The PCR product was then run on a 2% agarose gel containing SYBR-safe and visualized using the G.Box (Syngene) and Genesnap (Syngene).

### In vivo *Mtb infection and CFU analysis*

Anaesthetized C57BL/6 mice were infected intranasally with approximately 100 CFU/mouse in sterile saline (25 µl per nostril). Infected mouse lungs were homogenized to produce a single-cell suspension to determine lung CFU at 3- and 6-weeks post-infection and to determine bacilli uptake at 1-day post-infection. CFU assays were performed by plating whole lung homogenates on 7H11 agar plates containing 10% OADC and 0.5% glycerol for 2–3 weeks.

### Flow cytometry

Briefly, single-cell suspensions from the lung tissues were prepared by incubation in DMEM containing 0.18 mg/ml Collagenase Type I (Sigma, St. Louis, MO), 0.02 mg/ml DNase I (Sigma, St. Louis, MO) for 1 hour at 37°C under constant rotation, followed by mechanically passing through a 100 μm and 70 μm cell strainer sequentially. Erythrocytes were lysed using red blood cell (RBC) lysis buffer (155 mM NH_4_Cl, 12 mM NaHCO_3_, 0 · 1 mM EDTA). A single-cell suspension (1x10^6^ cells) from infected lung tissue was stained for the following surface markers suspended in FACs buffer comprising of PBS supplemented with 1% BSA and 0.1% NaN_3_: CD64 (Clone X54-5/7 PeCy7, BioLegend), Ly6C (Clone AL-21 PerCPCy5.5, BD Biosciences), CD11b (Clone M1/70 V450, BD Biosciences), MHCII (Clone M5/114.15.2 AF700, BioLegend), CD103 (Clone M290 PE, BD Biosciences), CD11c (Clone HL3 APC, BD Biosciences), SiglecF (Clone E5-2440 APC-Cy7, BD Biosciences), Ly6G (Clone 1A8 FITC, BD Biosciences), F4/80 (Clone BM8 PeCy7, eBiosciences), CD4 (Clone RM4-5 BV510, BD Biosciences), CD44 (Clone IM7 PE, BD Biosciences), CD3 (Clone 500A2 AF700, BD Biosciences), CD62L (Clone MEL-14 V450, BD Biosciences), CD19 (Clone 1D3 PerCPCy5.5, BD Biosciences) and CD8 (Clone 53–6.7 APC, BD Biosciences). The acquisition of samples was conducted using BD LSR Fortessa and the gating strategies outline in the supplementary materials (Fig. S4 and S5). Data analysis was performed with FlowJo v10 software (Treestar, Ashland, OR, US)

### Histology and alveolar space assessment

Infected murine lungs were excised, fixed using 4% phosphate-buffered formalin solution and stained with hematoxylin and eosin as previously published [36]. Four individual sections were taken per mouse for downstream analysis via histology. Analysis of lung sections and assessment of alveolar space was performed using NIS advanced software on a Nikon (Tokyo, Japan) 90i microscope.

### The enzyme-linked immunosorbent assay

The supernatants extracted from the whole lung homogenates were isolated from infected murine lungs via centrifugation. Filtered supernatants were used to detect IL-12p40, IFN-γ, IL-6, CCL2, GM-CSF, TGFβ, IL-4 (BD Biosciences), G-CSF, GM-CSF, CXCL2, CCL3, CCL5, CXCL1, CXCL5, CXCL10, IL-1β (R&D Scientific), IL-1α, IL-1β, TNF, IL-17, IL-10, IFN-β, and IL-23 (BioLegend) via the enzyme-linked immunosorbent assay (ELISA) according to manufacturer suggested dilutions. Chemokine and cytokine protein expression was measured using SoftMax Pro 6.

### Statistical analysis

All experimental data were analyzed using Graph-Pad Prism 8.0.2, one-way ANOVA was used. A *P value of less 0.05 was considered significant, with *P < 0.05, **P < 0.01, ***P < 0.001 and ****P < 0.0001. Sample size determined using G*Power (version 3.19.7). Optimally, a total sample size of 18 (n = 6 per group) with an expected effect size (*f*= 1.5) and an α = 0.5 allowed for a statistical power of 99%. Alternatively, a minimum sample size of 9 (n = 3 per group) allowed for 87%.

## Results

### Mtb amidase 1 and 4 deletion mutants induce an elevated proinflammatory response

**Figure 1.**
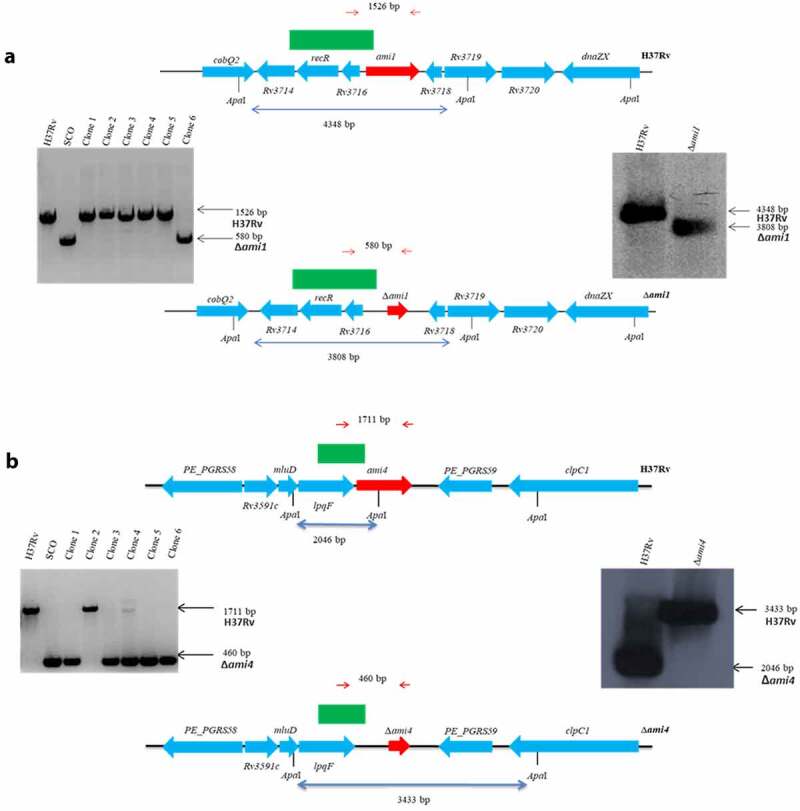
Genotypic analysis of the amidase Mtb strains by PCR and Southern blot analysis. The genomic map of the relevant locus is shown for the (a) H37RvΔ*ami1* strain, (b) H37RvΔ*ami4* strain and the wild-type H37Rv strain. Also shown on the left is the PCR confirmation of the genotype and Southern blotting is shown on the right. For PCR confirmation of ami1 and ami4, chromosomal DNA was used to amplify the ami1 alleles from the wild type and mutant strains using the primers described in Table S1 and indicated as red arrows above. The expected sizes of the amplicons are as follows: *ami1/*Rv3717:1526 bp and Δ*ami1*: 580 bp; *ami4/*Rv3594, 1711 bp and Δ*ami4*, 460 bp. For the Southern blot analysis, chromosomal DNA from the parental and mutant strain was digested with ApaI. The probe used for hybridization is shown as a solid green box and the expected sizes are indicated by the blue arrows. The figures are not drawn to scale

**Figure 2.**
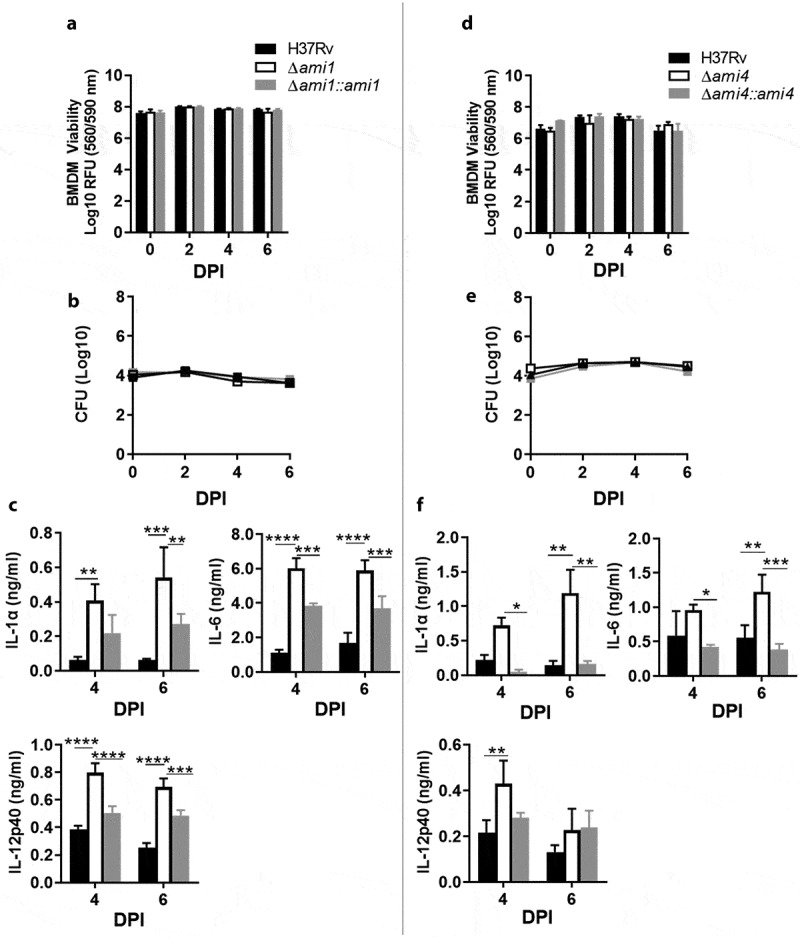
Mtb infection of IFN-γ-stimulated macrophages with the *Δami1* and *Δami4* mutant induced elevated proinflammatory responses with no effect on macrophage cell viability and intracellular Mtb growth. Cell viability of IFN-γ-stimulated (100 U/mL for ~12 h) BMDM infected with the (a) *Δami1* mutant, complemented strain (*Δami1::ami1*) and wild-type H37Rv and (b) CFU counts of BMDM infected with the *Δami1* mutant, complemented strain (*Δami1::ami1*) and wild-type H37Rv. Proinflammatory cytokine production from infected macrophages was measured via ELISA at 4- and 6-days post-infection for BMDMs infected with the (c) *Δami1* mutant, complemented strain (*Δami1::ami1*) and wild-type H37Rv. (d) Cell viability of IFN-γ-stimulated (100 U/mL for ~12 h) BMDM infected with the *Δami4* mutant, complemented strain (*Δami4::ami4*) and wild-type H37Rv was measured via Cell Titer Blue at 0 (4 hours), 2, 4 and 6 days post-infection. (e) CFU counts of BMDM infected with the *Δami4* mutant, complemented strain (*Δami4::ami4*) and wild-type H37Rv. (f) Proinflammatory cytokine production from infected macrophages was measured via ELISA at 4- and 6-days post-infection for BMDMs infected with the *Δami4* mutant, complemented strain (*Δami4::ami4*) and wild-type H37Rv. (*P ≤ 0.05, **P ≤ 0.01, ***P ≤ 0.001, ****P ≤ 0.0001, one-way ANOVA, n = 3). DPI = days post-infection. Data are representative of one (1A, 1D) and two (1B, 1 C, 1E and 1 F) experiments

Mutants defective for the Ami1 and Ami4 genes were generated using two-step allelic exchange mutagenesis and genotyped by PCR and Southern blot (Figure 1(a,b)). To evaluate if Mtb amidases regulate host immune responses, IFN-γ-stimulated murine macrophages were infected with the Δ*ami1* and Δ*ami4* Mtb mutants. Using Cell Titer Blue assay, we observed that the cell viability of murine bone marrow-derived macrophages (BMDM) was not affected following infection with either Δ*ami1* and Δ*ami4* Mtb mutants, when compared to the wild-type Mtb H37Rv and genetically complemented strains (Figure 2(a,d)). Furthermore, both mutants were equally sensitive to the IFN-γ-induced antimicrobial activity of BMDM relative to the wild-type and genetically complemented strain as measured by classical colony-forming unit (CFU) enumerations (Figure 2(b,e)). Infection of BMDM with the Δ*ami1* mutant induced a prominently proinflammatory cytokine response, through increased secretion of IL-1α, IL-6, and IL-12p40 (Figure 2(c)). Similarly, infection of BMDM with the Mtb Δ*ami4* mutant resulted in significantly increased production of IL-1α, IL-6, and IL-12p40 (Figure 2(f)). Whilst the bacterial burden in IFNγ-stimulated macrophages remained similar between the H37Rv and amidase knockout strains (Figure 2(b,e)), increased secretion of proinflammatory cytokines was observed in macrophages infected with the Ami mutants relative to H37Rv (Figure 2(c,f)). The immune responses of naïve macrophages (M0) infected with Ami1 and Ami4 Mtb mutant strains relative to complemented and WT controls were also assessed. Similar to IFN-γ stimulated macrophages, the Δ*ami1* strain significantly induced the secretion of IL-1α and IL-6 at 6-DPI and IL-12p40 at 4-DPI when compared to H37Rv (Fig S1C). Naïve macrophages infected with the Δ*ami4* mutant also significantly increased the secretion of IL-1α and IL-6 when compared to H37Rv (Fig. S1F). IL-12p40 remained unchanged at 4- and 6-DPI between the groups (Fig. S1F). Mycobacterial burden, as well as BMDM cell viability, were similar for both Δ*ami1* (Fig. S1A, B) and Δ*ami4* strains (Fig. S1D, E). Together, these results demonstrate that Ami1 and Ami4 are dispensable for growth in macrophages. However, the deletion of either Mtb amidases can induce an IFN-γ independent increased acute proinflammatory cytokine responses during macrophage infection.

### Ami1 deletion promotes cytokine/chemokine responses whilst simultaneously decreasing cell recruitment and improving lung pathology during acute infection in mice

**Figure 3.**
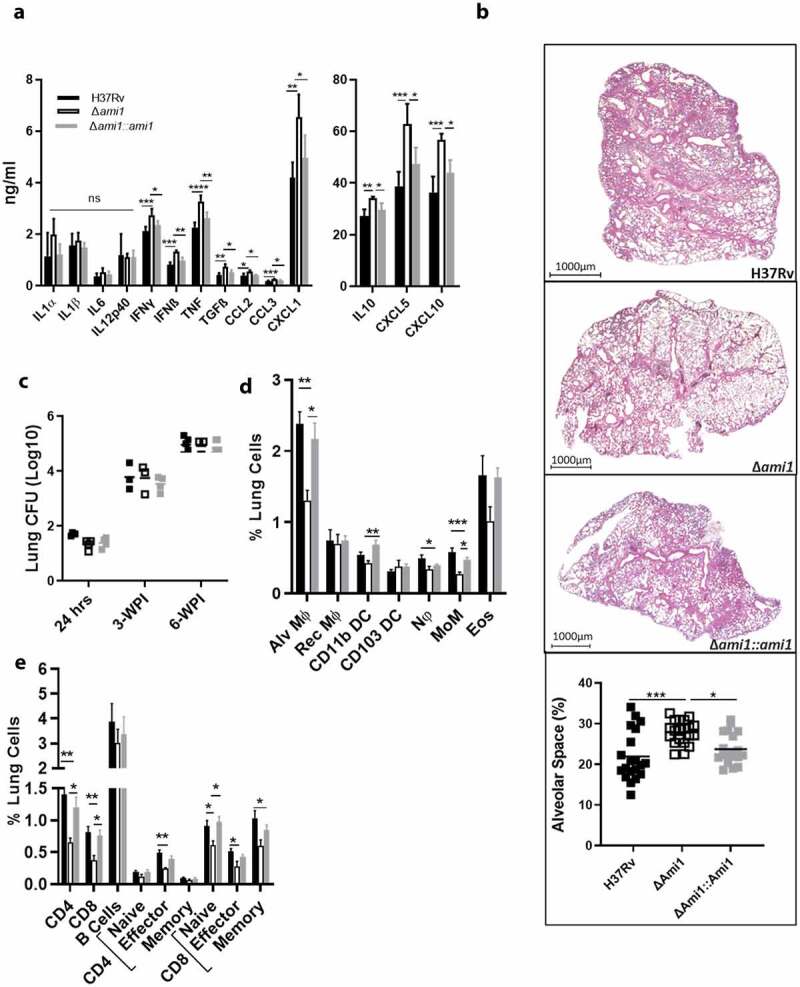
Infection of mice with the *Δami1* mutant results in an elevated cytokine/chemokine response at 3-WPI, reduced specific myeloid and lymphoid cell populations in the lung and reduced lung pathology. C57BL/6 mice were infected intranasally with 100 CFU/mouse of the Mtb Δ*ami1* mutant, complemented *Δami1::ami1* and WT, H37Rv. (a) Cytokine and chemokine levels from whole lung homogenates of Mtb-infected mice were measured by ELISA at 3-WPI. (b) H&E histology staining of Mtb-infected lungs at 3-WPI. Inflammation was quantified as a measure of alveolar space (Magnification 10x). Percentage of lung alveolar airspaces were quantified from 4 deep cuts of H&E lung sections per mice (30 μm apart). Each data point represents an individual cut. (C) Mice were sacrificed at 3- and 6-WPI to measure mycobacterial burden by CFU enumeration in the lungs. Infected mice were sacrificed at 3-WPI and lungs were collected to measure the frequency and cell numbers of (d) myeloid and (e) lymphoid cell populations. Alveolar macrophages (Alv MΦ) = CD64^+^SiglecF^+^CD11c^+^, recruited interstitial macrophages (Rec MΦ) = CD64^+^CD11c^−^SiglecF^+^, CD103 dendritic cells (DC) = MHCII^+^CD11c^+^CD103^+^CD11b^−^, CD11b DC = MHCII^+^CD11c^+^CD103^−^CD11b^+^, neutrophils (Nφ) = LY6G^+^CD11b^+^, monocytes (MoM) = CD64^+^ CD11b^+^CD11c^+^, eosinophils (Eos) = CD64^−^SiglecF^+^CD11b^+^, B cells = CD19^+^CD3^−^, CD8^+^ T cells = CD3^+^CD4^−^CD8^+^, CD4^+^ T cells = CD3^+^CD4^+^CD8^−^, naïve T cells = CD62L^+^CD44^+^, memory T cells = CD62L^+^CD44^−^ effector T cells = CD62L^−^CD44^+^ (*P ≤ 0.05, **P ≤ 0.01, ***P ≤ 0.001, ****P ≤ 0.0001, one-way ANOVA, n = 5–6). Data in panels A to E are representative of two independent experiments

To investigate if the elevated proinflammatory responses elicited by the amidase mutants during macrophage infection translated to elevated immune responses *in vivo*, we infected C57BL/6 mice intranasally with the Δ*ami1* or Δ*ami4* Mtb mutant strains. In the acute phase, at 3-weeks post-infection (WPI), the Δ*ami1* mutant significantly increased the production of several proinflammatory cytokines (IFN-γ and TNF), type 1 interferon (IFN-β), regulatory cytokines (TGFβ and IL-10), and chemokines (CCL2, CCL3, CXCL1, CXCL5, and CXCL10) in whole lung homogenates when compared to the Δ*ami1* complemented and wild-type strains (Figure 3(a)). Of interest, Mtb Δ*ami1* mutant displayed reduced pulmonary histopathology, as measured by percentage alveolar space relative to the Δ*ami1* complemented and wild-type strains (Figure 3(b)). Despite the increased inflammatory cytokine and chemokine responses at 3-WPI, the mycobacterial growth rates of Δ*ami1* in the lungs were comparable to the wild-type strain as measured by classical CFU enumerations at 3-weeks post-infection (Figure 3(c)).

Using flow cytometry, we measured numerous immune cell populations within the lungs at 3-weeks post-infection. Consistent with histological analysis, we determined that infection with Δ*ami1* significant decreased recruitment of alveolar macrophages, CD11b^+^ DC, neutrophils and monocytes (Figure 3(d)) with a concomitant decrease in total CD4^+^ T cells, CD8^+^ T cells, effector CD4^+^ T cells as well as naïve, effector and memory CD8^+^ T cells (Figure 3(e)), when compared to WT Mtb. Within the mediastinal lymph nodes (LN), at 3-WPI, the Mtb Δ*ami1* mutant, infection resulted in an increased frequency of B cells (Fig. S1C). However, when calculated for total cell numbers within the lung and mediastinal lymph nodes, no measurable difference in cellular recruitment was observed between the groups (Fig. S2A, B, D). Taken together, these data indicate that, whilst Ami1 is dispensable for the colonization of the lung during infection, it is important for the reduction of cytokine responses and inflammation during acute host infection. Furthermore, the deletion of Ami1 can alter host immune cell recruitment within the lung and mediastinal lymph node at 3-WPI.

### Ami4 knockout does not affect host immune responses or lung pathology during acute infection in mice

**Figure 4.**
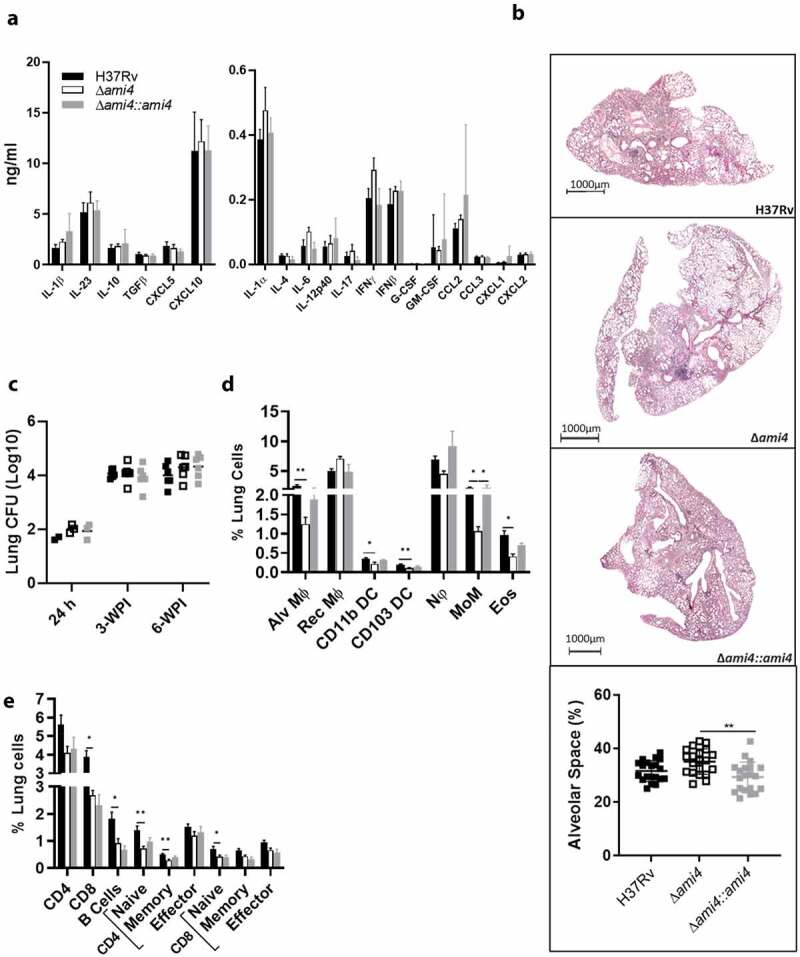
Mtb Ami4 is dispensable for the modulation of the host immune response during acute and chronic-phase infection and has no effect on lung pathology in mice. C57BL/6 mice were infected intranasally with 100 CFU/mouse of the Mtb Δami4 mutant, complemented Δami4::ami4 and WT, H37Rv. (a) Cytokine and chemokine levels from whole lung homogenates of Mtb-infected mice were measured by ELISA at 3-WPI. (b) H&E histology staining of Mtb-infected lungs at 3-WPI. Inflammation was quantified as a measure of alveolar space (Magnification 10x). Percentage of lung alveolar airspaces were quantified from 4 deep cuts of H&E lung sections per mice (30 μm apart). Each data point represents an individual cut. (c) Mice were sacrificed at 3- and 6-WPI to measure mycobacterial burden by CFU enumeration in the lungs. Infected mice were sacrificed at 3-WPI and lungs were collected to measure the frequency and cell numbers of (d) myeloid and (e) lymphoid cell populations. Alveolar macrophages (Alv MΦ) = CD64^+^SiglecF^+^CD11c^+^, recruited interstitial macrophages (Rec MΦ) = CD64^+^CD11c^−^SiglecF^+^, CD103 dendritic cells (DC) = MHCII^+^CD11c^+^CD103^+^CD11b^−^, CD11b DC = MHCII^+^CD11c^+^CD103^−^CD11b^+^, neutrophils (Nφ) = LY6G^+^CD11b^+^, monocytes (MoM) = CD64^+^ CD11b^+^CD11c^+^, eosinophils (Eos) = CD64^−^SiglecF^+^CD11b^+^, B cells = CD19^+^CD3^−^, CD8^+^ T cells = CD3^+^CD4^−^CD8^+^, CD4^+^ T cells = CD3^+^CD4^+^CD8^−^, naïve T cells = CD62L^+^CD44^+^, memory T cells = CD62L^+^CD44^−^ effector T cells = CD62L^−^CD44^+^ (*P ≤ 0.05, **P ≤ 0.01, ***P ≤ 0.001, ****P ≤ 0.0001, one-way ANOVA, n = 5–6). Data in panels A to E are representative of one experiment

We further investigated the acute host immune response to Mtb Ami4 deficiency using the Δ*ami4* mutant. The mycobacterial burden (Figure 4(c)), lung tissue inflammation as measured by percentage free alveolar space (Figure 4(b)) and cytokine/chemokine expressions (Figure 4(a)) were similar between Δ*ami4* and the wild-type control. In the lung, at 3-WPI, Ami4 deletion elicited decreased percentages of alveolar macrophages, CD11b^+^ DC, CD103^+^ DC, monocytes and eosinophils (Figure 4(d)). Additionally, within the lung, a reduction in CD8^+^ T cells, B cells, naïve & memory CD4^+^, as well as a reduction in naïve CD8^+^ T cells, was observed when compared to WT Mtb (Figure 4(e)). Further, within the lymph nodes at 3-WPI, Ami4 deletion elicited decreased naïve and memory CD4^+^ and CD8^+^ T cells by percentages when compared to WT Mtb (Fig. S3C). However, when calculated for total cell numbers within the lung and mediastinal lymph nodes, no measurable difference in cellular recruitment was observed between the groups. These *in vivo* data indicate that whilst Ami4 deletion resulted in a slightly altered cellular recruitment by percentages, the functionality of the host immune response remained unaltered relative to wild-type controls as measured by cytokine/chemokine responses. Hence, these data demonstrate that Ami4 deletion has a negligible effect on the host immune response as both cellular recruitment, cytokine/chemokine secretion and histopathology were similar. Further, the CFU data illustrate that Ami4 is dispensable for the colonization of the lung during infection.

## Discussion

Cell wall amidases cleave the covalent bonds responsible for the stabilization of the cell wall sacculus and thus represent autolytic enzymes that can be targeted for drug therapy. Most research characterizing the intracellular function of amidases has been conducted in *E. coli* or *M. smegmatis*. In the present study, we sought to elucidate the effect of amidase knockout on the host immune response to Mtb infection. Despite the similarity of the crystal structures [27,28] and genetic conservation between multiple diverse species ranging from bacteria to fungi [37], few studies have fully assessed loss-of-function effects of amidases on the host immune response to mycobacterial amidase mutants [33,38]. Emerging evidence suggests that these enzymes may play variable and essential roles in mycobacterial homeostasis – especially during stress, such as has been observed in Ami2 [29]. In comparison to recent published Ami mutant strains of *M. smegmatis*, our Mtb data indicate the probability of alternative roles of Ami1 and Ami4 in pathogenic vs. nonpathogenic mycobacterial species. For example, the knockout of Ami1 in *M. smegmatis* resulted in defective bacterial fission, evident through the formation of ectopic lateral buds, mislocalization of the elongation apparatus, bundling of the FtsZ ring and defective septal PG turnover [32]. Further, the Ami1 knockout of *M. smegmatis* displayed higher cell wall permeability and was more sensitive to several antibiotics. Contrary to our *in vitro* macrophage infection studies with Mtb amidase mutants, it is clear that Ami1 promotes the bacterial survival of *M. smegmatis* within macrophages possibly through Ami1-mediated reduction of host inflammation [39]. These differences could be potentially attributed to the induction of distinct host-immune responses within macrophages infected between nonpathogenic *M. smegmatis* and pathogenic *M. tuberculosis* [40].

In a recent *in vivo* infection study, C57BL/6 mice were infected with an Ami1 deficient *M. smegmatis* mutant. Those authors displayed a diminished uptake of the mutant strain within the lungs, thus confirming the worsened invasion ability of these mutants observed *in vitro* [38]. In this case, deletion of Ami1 led to less tissue inflammation despite residing within the lungs and twice as long relative to the wild-type and complemented strains before being cleared. Interestingly, this finding was recently verified in the Mtb model by Healy et al. [33], where Ami1 was found to be important to prolong chronic Mtb infection [33]. This observation contrasts with our data, where at 6-weeks post-infection (42-days post), the bacterial burden in mice infected with the Mtb Ami1 mutant relative to WT was similar. This disparity may be a result of the differences in the timepoints being assessed between the two studies (6-WPI vs. 8-WPI). Healy et al. [33], observed a significant reduction in the Mtb Ami1 mutant CFU at 8-WPI relative to WT. It is possible that, with further 2 weeks of infection, we may have observed the same CFU dynamics, which indicates that Ami1 is required to prolong Mtb infection *in vivo*. Further, the 6-WPI timepoint examined in this study may be a transitionary period from acute to chronic-phase infection. Consistent with Healy et al. [33] findings, however, our data confirm that both naïve and interferon-gamma activated macrophages have an unchanged ability to clear the Ami1 mutant relative to the WT control. Furthermore, we also found that Ami1 is dispensable for the intracellular growth of Mtb in mice at similar time points. However, we show for the first time that the single gene deletion of Ami4 may not affect the ability of *M. tuberculosis* to colonize macrophages and mice.

Wild-type Mtb bacilli which proliferate intracellularly within the phagosome of macrophages have been observed to exhibit filamentous bacterial chains and a dysregulated FtsZ ring phenotype [41]. Interestingly, similar phenotypes have been observed in *Salmonella enterica* where the authors assessed the role of the AmiA and AmiC knockout – conserved orthologues of mycobacterial Ami1 and Ami4 [42]. They provided evidence showing that bacilli which were grown under low pH, high osmolarity and sublethal antimicrobial stress formed chains of cells that were more sensitive to the host immune response. Further, within the nonpathogenic *M. smegmatis* model of infection chaining of daughter cells was observed in bacilli deficient for Ami1 [32]. Contrasting to these studies, Healy et al. [33] reported that Ami1 deficiency in Mtb induced no filamentation/chaining in the Mtb Ami1 mutant relative to wild-type controls [33]. This result supports our data where the bacterial burdens observed between wild-type and amidase-deficient Mtb mutants were similar during acute and chronic-phase infection. Together, these data imply that the invasion handicap observed in the *M. smegmatis* Ami mutant does not occur in the Mtb model of infection. Additionally, Healy et al. [33] tested the Mtb Ami1 deletion mutant for atypical cell division phenomena. Those authors found that under regular culture conditions, Ami1 deletion did not affect the length of daughter cells nor the number of division-septa they contained [33].

Here, we report that bacterial burden in IFN-γ-stimulated macrophages remained similar between the H37Rv and Ami knockout strains whilst increased secretion of proinflammatory cytokines was observed in macrophages infected with the Ami mutants relative to H37Rv. Concurrent with this finding, bacterial burdens within the naïve macrophages remained unchanged whilst Ami1/4 deletion elicited a similar increase in proinflammatory cytokines. Taken together, these data indicate an IFN-γ independent increase in cytokines, which is likely mediated via Ami1 and Ami4 deletion in Mtb. These changes can likely alter cellular recruitment and activation during infection. Further, we observed that during the acute phase of infection with the Ami1 mutant resulted in less inflammation by immunohistochemistry, despite the detection of elevated proinflammatory cytokines and chemokines relative to the complemented and wild-type strains. Despite the decreased percentages of myeloid and lymphoid cells in the lungs (Figure 3), these data suggest that several of these cell types, which may induce the enhanced secretion of cytokines in Mtb Ami1 mutant infected group, may exhibit effector phenotypes. Additionally, Senzani et al. [32] showed that an Ami1 *M. smegmatis* mutant extruded cell wall material into the cytoplasm during binary fission. This phenomenon may account for the enhanced acute immune response observed *in vitro* and *in vivo* within this study, whilst maintaining the bacterial burden in an unknown mycobacterial-specific manner. Previous studies have highlighted the importance of NOD2 in the detection and immune response to mycobacterial antigen [43,44]. Certainly, the increased presence of PG muropeptide subunits, in the milieu has the potential to bind to NOD-like receptors and subsequently induce an enhanced proinflammatory cytokine response via canonical pathways. Several studies have highlighted the role of amidases in the recycling of PG fragment in gram-negative bacteria as well as in Mtb during growth and division [28,45]. Further, these studies have highlighted the potential impact that PG recycling has on the survival of the bacterium and the subsequent host immune response. The loss of Mtb-specific Ami1 or Ami4 was hypothesized to increase in the presence of such PG fragments, and thus, induce an elevated inflammatory signature in the host. Our data indicate that this may be occurring during acute infection. Further, our *in vivo* data indicate that the loss of Ami1 is more immunomodulatory than Ami4. This may explain the increased proinflammatory immune response observed at 3-weeks post-infection, despite the lack of tissue inflammation. However, whilst statistically significant, many of the changes noted in cytokine and chemokine secretion are minimal (1.1–1.7-fold) and may be only of slight biological significance. This is further illustrated by the unchanged bacterial burden observed during acute and chronic infection. However, the differences observed in cellular recruitment and tissue inflammation show that, though small, the deletion of Ami1 can increasingly alter the host immune response during acute infection relative to Ami4.

In conclusion, we provide evidence describing the host immune response, both *in vitro* and *in vivo*, to two Mtb amidase knockouts, where the Δ*ami1* mutant induced a largely proinflammatory immune response both *in vitro* and at 3-WPI *in vivo*, relative to WT controls. Neither Ami1 nor Ami4 deletion resulted in a reduction of bacterial burdens within the lungs during acute (3-WPI) and chronic phase (6-WPI) infection. Future work interrogating the effect of multi-amidase-knockout mutants may confer more understanding.

## Supplementary Material

Supplemental Material

## Data Availability

The data that support the findings of this study are openly available in Mendeley Data at http://dx.doi.org/10.17632/62vpdns428.1
