# Supplementary material for: Deletion of N-acetylmuramyl-L-alanine amidases alters the host immune response to Mycobacterium tuberculosis infection
Source: Virulence. 2021 May 13;12(1):1227–38. doi: 10.1080/21505594.2021.1914448 (PMC8128173; doi:10.1080/21505594.2021.1914448)
Supplement: Supplemental Material [file KVIR_A_1914448_SM3969.docx]

**Deletion of N-acetylmuramyl-L-alanine amidases alters the host immune response to *Mycobacterium tuberculosis* infection**

***Supplementary Material***

Nathan Scott Kieswetter^1,2^, Mumin Ozturk^1,2^, Shelby-Sara Jones^1,2^, Sibusiso Senzani^4†^, Melissa Dalcina Chengalroyen^4‡^, Frank Brombacher^1,2,3^, Bavesh Kana^4^, and Reto Guler^1,2,3 *^

^1^International Centre for Genetic Engineering and Biotechnology, Cape Town Component, Cape Town 7925, South Africa.

^2^Department of Pathology, University of Cape Town, Institute of Infectious Diseases and Molecular Medicine (IDM), Division of Immunology and South African Medical Research Council (SAMRC) Immunology of Infectious Diseases, Faculty of Health Sciences, University of Cape Town, Cape Town 7925, South Africa.

^3^Wellcome Centre for Infectious Diseases Research in Africa (CIDRI-Africa), Institute of Infectious Disease and Molecular Medicine (IDM), Faculty of Health Sciences, University of Cape Town, Cape Town 7925, South Africa.

^4^DSI/NRF Centre of Excellence for Biomedical TB Research, Faculty of Health Sciences, University of the Witwatersrand, National Health Laboratory Service, Johannesburg, 2001, South Africa.

^†^ Present address: Department of Medical Microbiology, University of KwaZulu-Natal, KwaZulu-Natal, South Africa.

^‡^ Present address: Molecular Mycobacteriology Research Unit, University of Cape Town, Cape Town, South Africa.

****Correspondence***: Tel: +27-21-4066033; Fax: +27-86-6407594 ***E-mail***: [reto.guler@uct.ac.za](mailto:reto.guler@uct.ac.za)

***Disclosure***: The authors declare no conflict of interest.


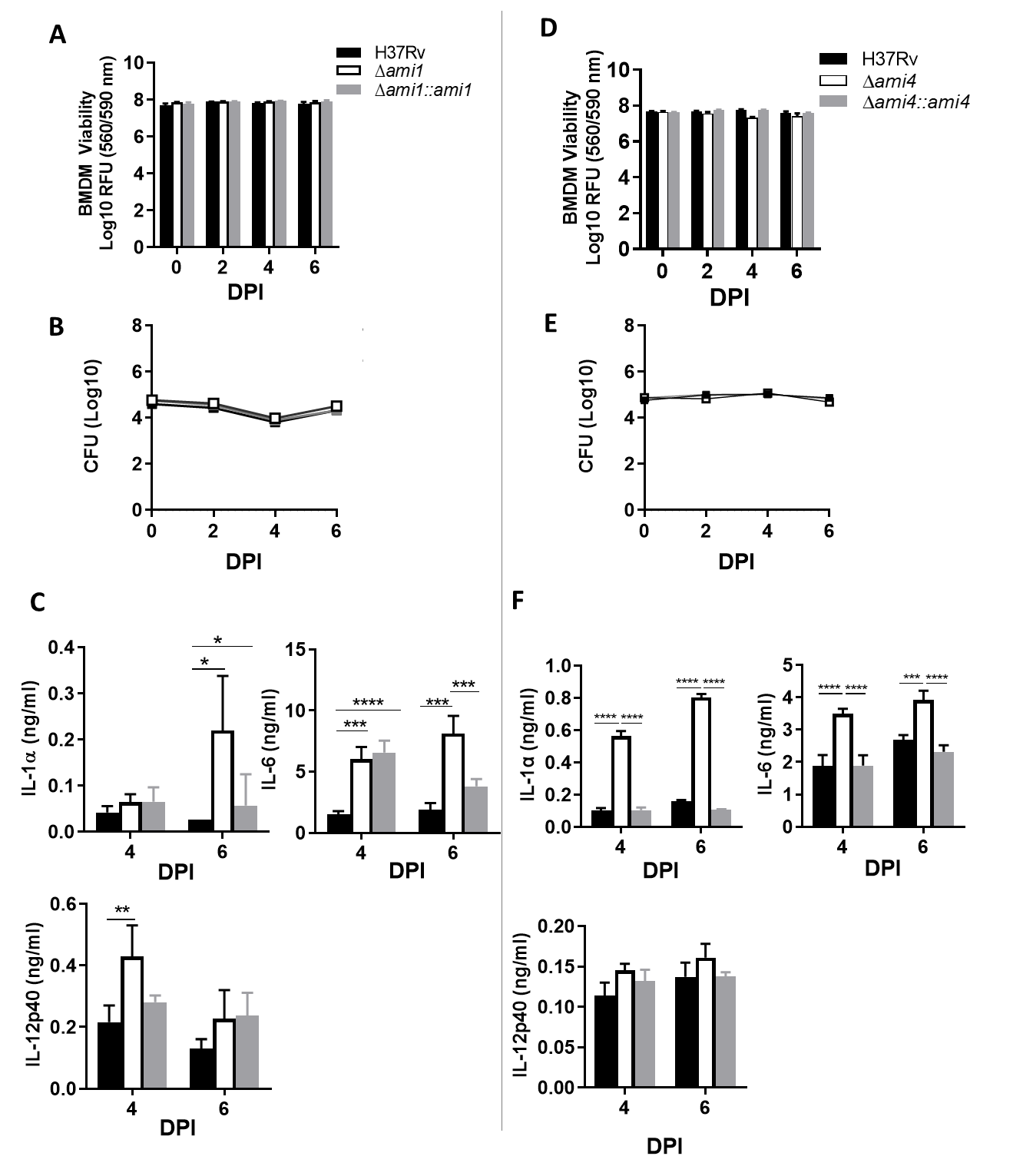


Figure S1: **Mtb infection of naïve macrophages with the *Δami1* and *Δami4* mutant induced elevated proinflammatory responses with no effect on macrophage cell viability and intracellular Mtb growth.** Cell viability of naïve BMDM infected with the **A**) *Δami1* mutant, complemented strain (*Δami1*::*ami1*) and wild-type H37Rv and **B**) CFU counts of BMDM infected with the *Δami1* mutant, complemented strain (*Δami1*::*ami1*) and wild-type H37Rv. Proinflammatory cytokine production from infected macrophages was measured via ELISA at 4- and 6-days post-infection for BMDMs infected with the **C)** *Δami1* mutant, complemented strain (*Δami1*::*ami1*) and wild-type H37Rv**. D)** Cell viability of naïve BMDM infected with the *Δami4* mutant, complemented strain (*Δami4*::*ami4*) and wild-type H37Rv was measured via Cell Titer Blue at 0 (4 hours), 2, 4 and 6 days post-infection. **E**) CFU counts of BMDM infected with the *Δami4* mutant, complemented strain (*Δami4*::*ami4*), and wild-type H37Rv. **F**) Proinflammatory cytokine production from infected macrophages was measured via ELISA at 4- and 6-days post-infection for BMDMs infected with the *Δami4* mutant, complemented strain (*Δami4*::*ami4*), and wild-type H37Rv. (*P ≤ 0.05, **P ≤ 0.01, ***P ≤ 0.001, ****P ≤ 0.0001, one-way ANOVA, n=3). DPI=days post-infection. Data in panels A to F are representative of one experiment.

**
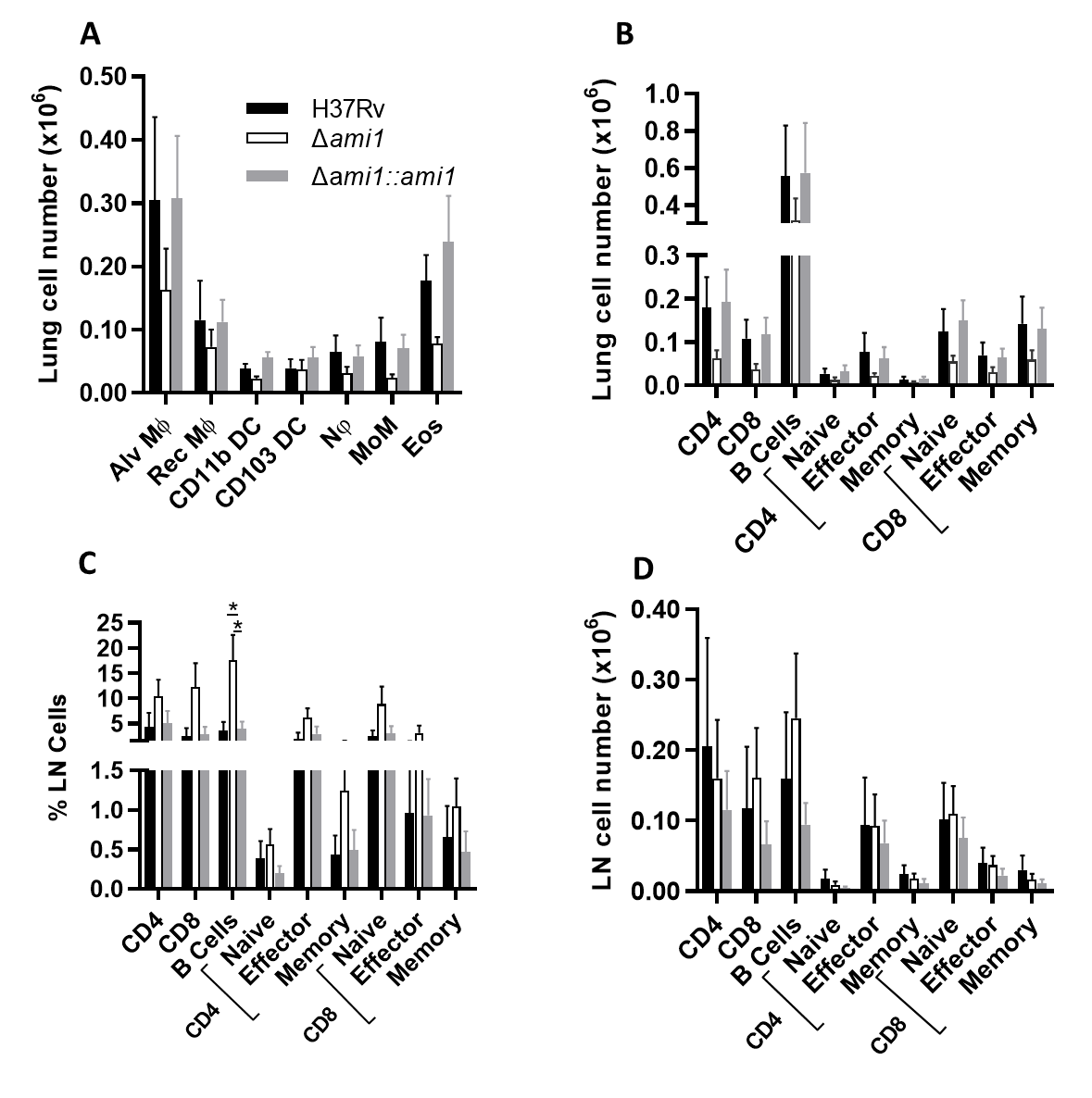
**

Figure S2: **At 3-WPI, Δ*ami1* mutant infection in mice reduces specific myeloid and lymphoid cell populations in the lung whilst recruiting B cells in mediastinal lymph nodes.** C57BL/6 mice were infected intranasally with 100 CFU/mouse of Δ*ami1* mutants, Δ*ami1::ami1* and wild-type H37Rv. Infected mice were sacrificed at 3-WPI and lungs were collected to measure **A)** myeloid cell number, **B**) lymphoid cell number. **C, D)** Mediastinal lymph nodes were collected to determine the frequency and cell numbers of lymphoid populations. Alveolar macrophages (Alv MΦ) = CD64^+^SiglecF^+^CD11c^+^, recruited interstitial macrophages (Rec MΦ) = CD64^+^CD11c^-^SiglecF^+^, CD103 dendritic cells (DC) = MHCII^+^CD11c^+^CD103^+^CD11b^-^, CD11b DC = MHCII^+^CD11c^+^CD103^-^CD11b^+^, neutrophils (Nφ) = LY6G^+^CD11b^+^, monocytes (MoM) = CD64^+^ CD11b^+^CD11c^+^, eosinophils (Eos) = CD64^-^SiglecF^+^CD11b^+^, B cells = CD19^+^CD3^-^, CD8^+^ T cells = CD3^+^CD4^-^CD8^+^, CD4^+^ T cells = CD3^+^CD4^+^CD8^-^, naïve T cells = CD62L^+^CD44^+^, memory T cells = CD62L^+^CD44^-^ effector T cells= CD62L^-^CD44^+^ (*P ≤ 0.05, one-way ANOVA, n=5-6). Data in panels A to D are representative of two independent experiments.


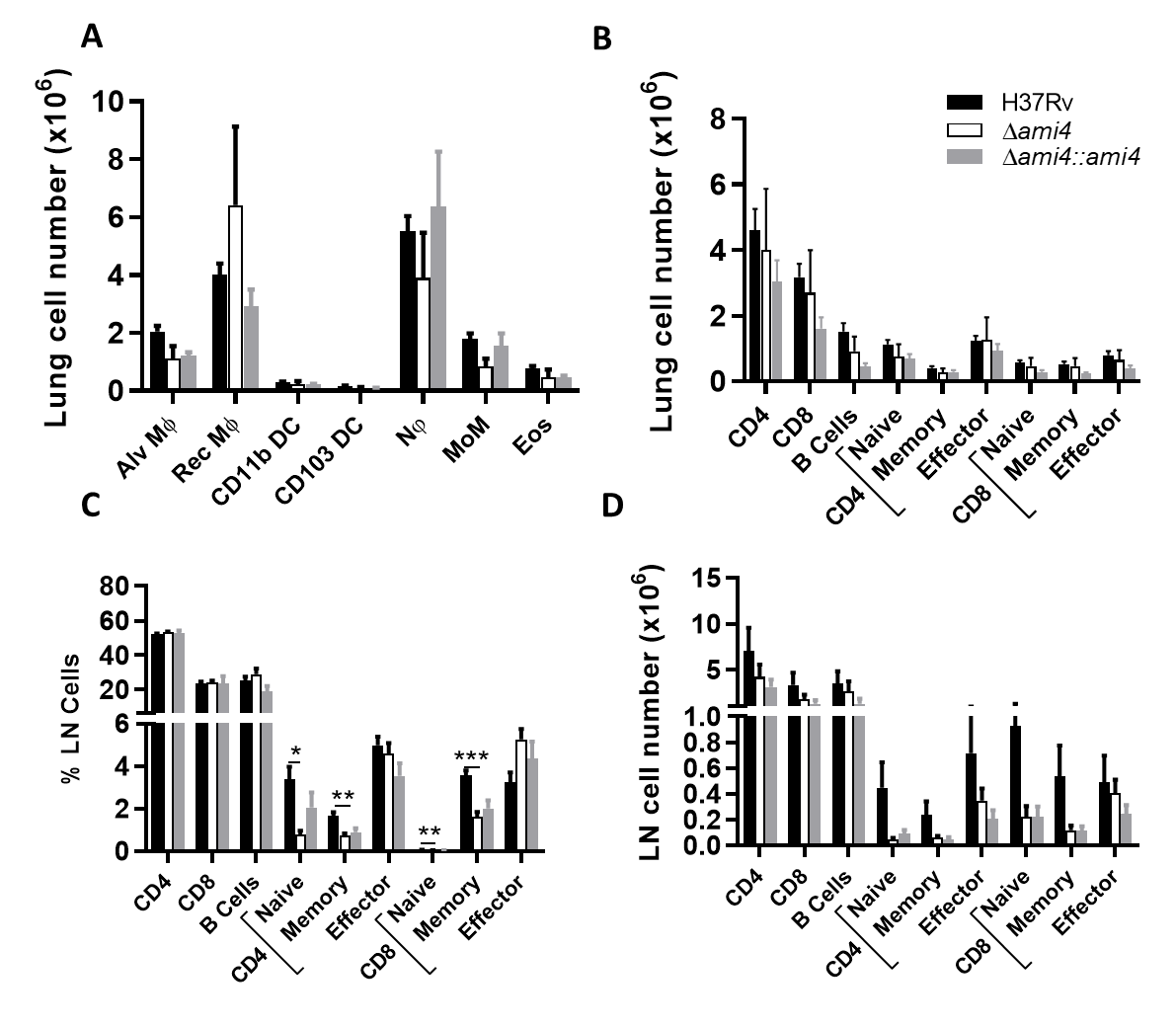


Figure S3**: At 3-WPI, Δ*ami4* mutant infection in mice reduces specific cell populations within the lung and the mediastinal lymph nodes.** C57BL/6 mice were infected intranasally with 100 CFU/mouse of the *Δami4* mutant, *Δami4*::*ami4* and wild-type H37Rv. Infected mice were sacrificed at 6-WPI and lungs were collected to measure **A)** myeloid cell number, **B**) lymphoid cell number. **C, D)** Mediastinal lymph nodes were collected to determine the frequency and cell numbers of lymphoid populations. Alveolar macrophages (Alv MΦ) = CD64^+^SiglecF^+^CD11c^+^, recruited interstitial macrophages (Rec MΦ) = CD64^+^CD11c^-^SiglecF^+^, CD103 dendritic cells (DC) = MHCII^+^CD11c^+^CD103^+^CD11b^-^, CD11b DC = MHCII^+^CD11c^+^CD103^-^CD11b^+^, neutrophils (Nφ) = LY6G^+^CD11b^+^, monocytes (MoM) = CD64^+^ CD11b^+^CD11c^+^, eosinophils (Eos) = CD64^-^SiglecF^+^CD11b^+^, B cells = CD19^+^CD3^-^, CD8^+^ T cells = CD3^+^CD4^-^CD8^+^, CD4^+^ T cells = CD3^+^CD4^+^CD8^-^, naïve T cells = CD62L^+^CD44^+^, memory T cells = CD62L^+^CD44^-^ effector T cells= CD62L^-^CD44^+^ (*P ≤ 0.05, **P ≤ 0.01, ***P ≤ 0.001) one-way ANOVA, n=5-6). Data in panels A to D are representative of one experiment.


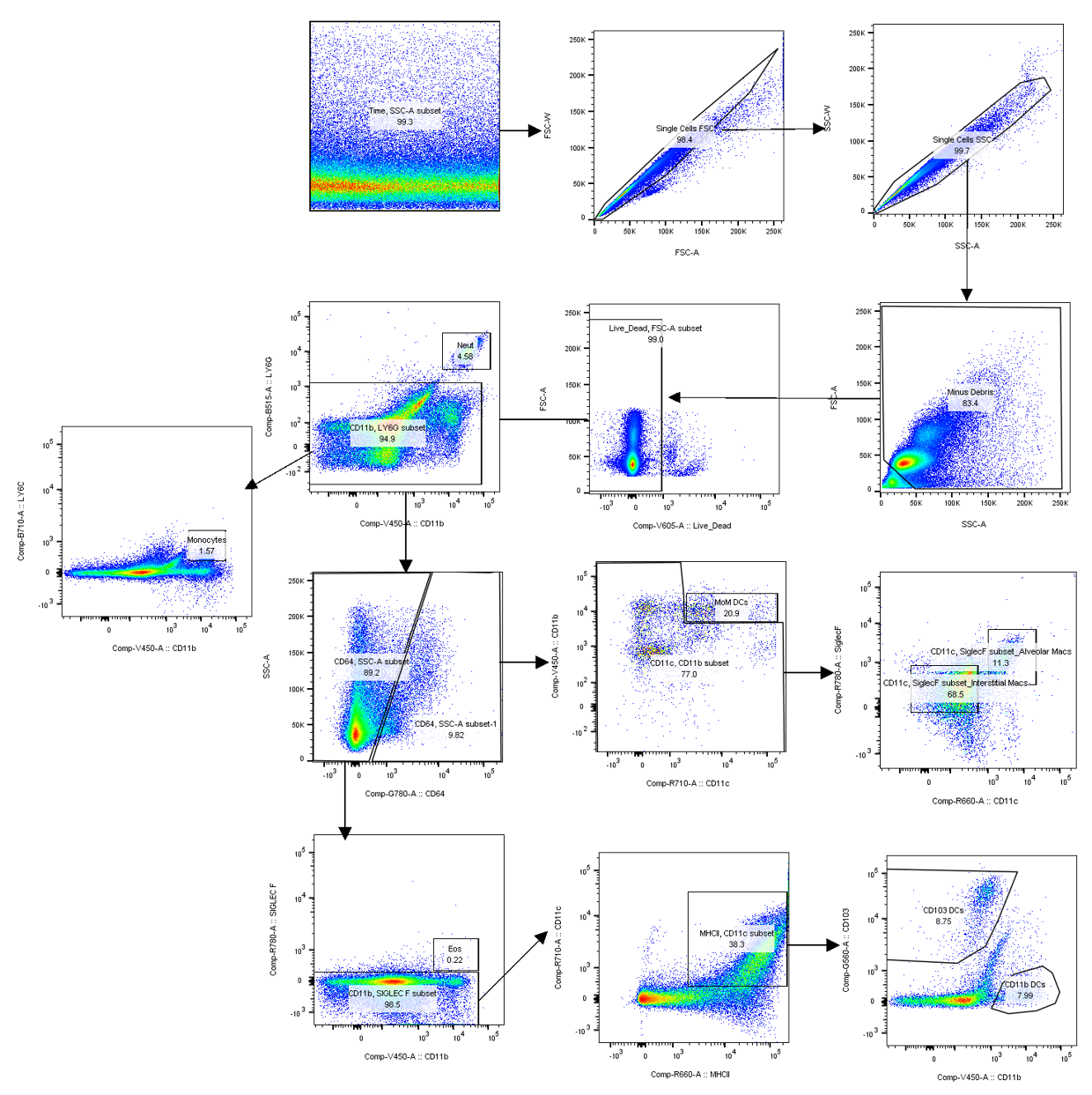


Figure S4: **Gating strategy for the identification of lung myeloid cell subsets.**


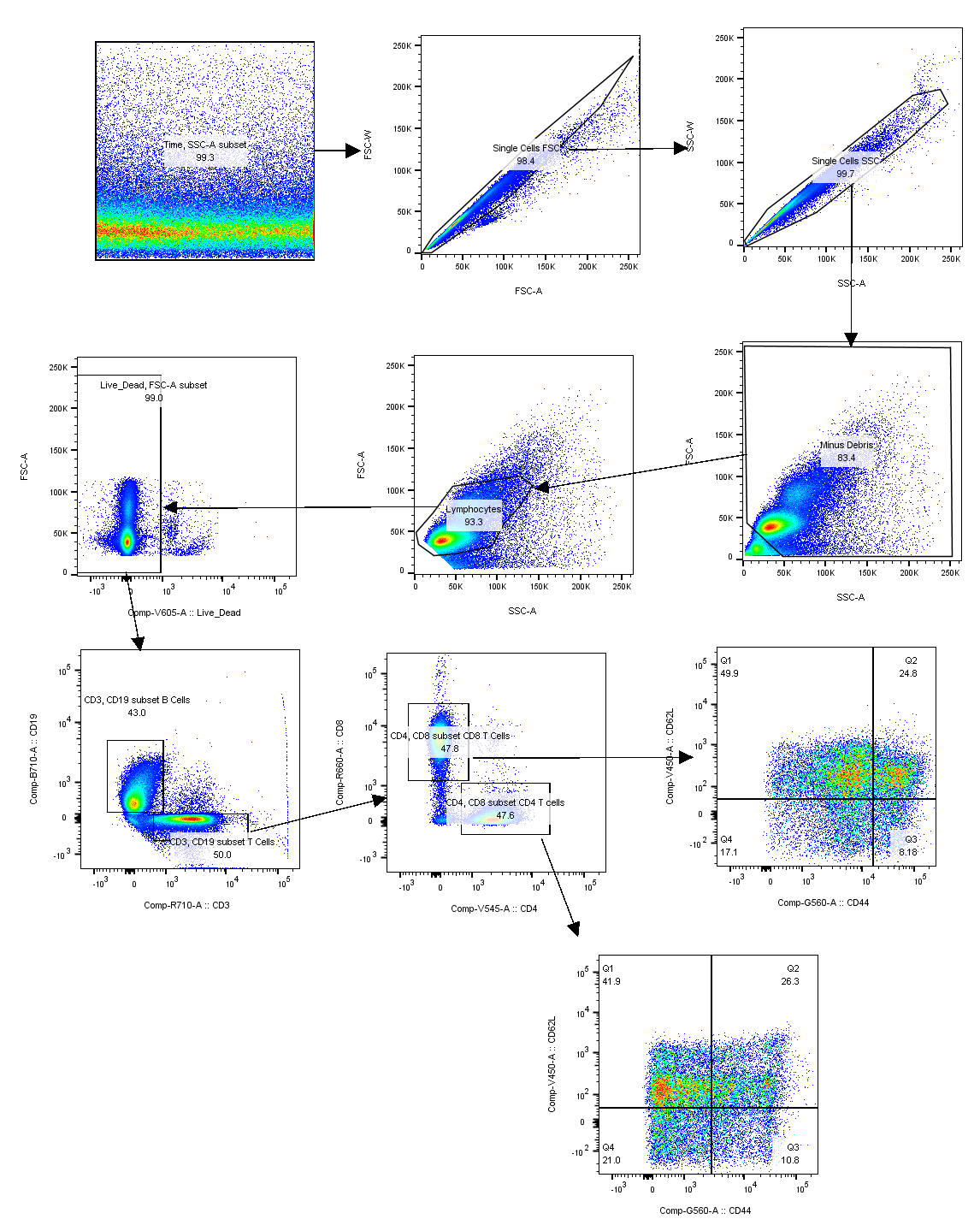


Figure S5: **Gating strategy for the identification of lung and lymph node lymphoid cell subsets.**

Table S1: **List of primers used in the construction of amidase deficient *M. tuberculosis* strains.**

*red: restriction endonuclease site

| **Primer** | **Sequence** | **Amplicon** |
| --- | --- | --- |
| H37*ami1*KOUSF | gtgaagcttgccgcattaccagctatgac | 1577 bp amplicon the 5’ region of the *ami1* gene and 96 bp of the *ami1* gene |
| H37*ami1*KOUSR | gtgtctagagtcgatgaagacgaccatgc |  |
| H37*ami1*KODSF | gtgtctagacgagggcaggcaaaaatac | 1579 bp amplicon the 3’ region of the *ami1* gene including 78 bp of the *ami1* gene |
| H37*ami1*KODSR | gtgggtaccgccatcaacctccagtagaca |  |
| H37*ami4*KOUSF | gtgaagcttACCGGCAAGACTGCATAAC | 1557 bp amplicon the 5’ region of the *ami4* gene and 39 bp of the *ami4* gene |
| H37*ami4*KOUSR | gtgtctagacacctcctcgagccaaatc |  |
| H37*ami4*KODSF | gtgtctagacgagctcggcaataaggtc | 1530 bp amplicon the 3’ region of the *ami1* gene including 82 bp of the *ami1* gene |
| H37*ami4*KODSR | gtgggtacccgatccgctgtgacaataga |  |
| H37*ami1*pMVF | gcgcgcgcaagcttgccatcttcgtcacctgc | 1126 bp *ami1* amplicon including 400 bp upstream the *ami1* start codon |
| H37*ami1*pMVR | gccgccgcgttaacCTAACGCGCCTGGCCCTG |  |
| H37*ami4*pMVF | gcgcgcgcaagcttCGGCCTCGCCCGTCCGAC | 1311 bp *ami4* amplicon including 400 bp upstream the *ami4* start codon |
| H37*ami4*pMVR | gccgccgcgttaacCCGGTTGACATCGTTGCA |  |

Table S2: **Bacterial strains and plasmids created/used in this study.**

| **Strain** | **Genotype** |
| --- | --- |
| H37Rv | Virulent laboratory isolate ATCC 25618 |
| H37∆*ami1* | Derivative of H37Rv carrying an unmarked, in-frame deletion in *ami1,* containing 96 bp of the 5’ and 78 bp 3’ regions, lacking 551 bp of the *ami1* gene |
| H37∆*ami4* | Derivative of H37Rv carrying an unmarked, in-frame deletion in *ami4,* containing 39 bp of the 5’ and 82 bp 3’ regions, lacking 707 bp of the *ami4* gene |
| **Plasmids** | **Genotype** |
| p2H37ΔAmi1G17 | Derivative of p2NIL carrying a truncated derivative of the H37Rv *ami1* gene and the *lacZ* and *sacB* genes from pGOAL17, Kan^R^ |
| p2H37ΔAmi4G17 | Derivative of p2NIL carrying a truncated derivative of the H37Rv *ami4* gene and the *lacZ* and *sacB* genes from pGOAL17, Kan^R^ |

Kan^R^: Kanamycin Resistance, Hyg^R^: Hygromycin Resistance
